# Quantitative evaluation on the characteristics of activated sludge granules and flocs using a fuzzy entropy-based approach

**DOI:** 10.1038/srep42910

**Published:** 2017-02-17

**Authors:** Fang Fang, Li-Li Qiao, Bing-Jie Ni, Jia-Shun Cao, Han-Qing Yu

**Affiliations:** 1 Key Laboratory of Integrated Regulation and Resource Development on Shallow Lakes, Ministry of Education, Hohai University, Nanjing 210098, China; 2 CAS Key Laboratory of Urban Pollutant Conversion, Department of Chemistry, University of Science & Technology of China, Hefei 230026, China

## Abstract

Activated sludge granules and flocs have their inherent advantages and disadvantages for wastewater treatment due to their different characteristics. So far quantitative information on their evaluation is still lacking. This work provides a quantitative and comparative evaluation on the characteristics and pollutant removal capacity of granules and flocs by using a new methodology through integrating fuzzy analytic hierarchy process, accelerating genetic algorithm and entropy weight method. Evaluation results show a higher overall score of granules, indicating that granules had more favorable characteristics than flocs. Although large sized granules might suffer from more mass transfer limitation and is prone to operating instability, they also enable a higher level of biomass retention, greater settling velocity and lower sludge volume index compared to flocs. Thus, optimized control of granule size is essential for achieving good pollutant removal performance and simultaneously sustaining long-term stable operation of granule-based reactors. This new integrated approach is effective to quantify and differentiate the characteristics of activated sludge granules and flocs. The evaluation results also provide useful information for the application of activated sludge granules in full-scale wastewater treatment plants.

Activated sludge process, after one century since its birth, is still at the center stage of wastewater treatment technologies and widely applied worldwide^1^. However, one major drawback of conventional activated sludge, typically in the form of flocs, is the loose structure, lower density and hence poor settling ability, which frequently results in poor effluent quality and high operating costs. In 1990’s, activated sludge in the form of granules were successfully cultured and exhibited excellent wastewater treatment performance. With denser structure and superior settling ability over the flocs, activated sludge granules enable higher level of biomass retention, more efficient treatment of high-strength wastewater, and better resistance to shock loadings, compared with the conventional activated sludge^2^,^3^,^4^,^5^. These benefits have stimulated increasing interests in optimizing and applying activated sludge granules as a new wastewater treatment technology. Soon, an excellent nutrient removal ability of granules was also found. Because of the formation of an anoxic zone in the granule center as a result of the oxygen transfer limitation, simultaneous carbon and nitrogen removal can be achieved and easily controlled^6^. In addition, simultaneous nitrogen and phosphorus removal could be achieved by granules under sequencing batch reactor (SBR) operating mode. Some denitrifying phosphate-accumulating organisms in the anoxic core of granules can utilize nitrite and nitrate, instead of oxygen, as an electron acceptor to drive phosphorus uptake under the anoxic and carbon-source-limiting conditions^4^,^7^,^8^,^9^. As thus, a simultaneous carbon, nitrogen and phosphorus removal can be achieved in a granule-based SBR under appropriate operating conditions^4^,^7^,^10^,^11^.

Despite of the superior pollutant removal ability, however, activated sludge granules frequently suffer from poor stability, making the practical application of aerobic granules challenging^12^. Furthermore, the limited transfer of substrate and oxygen within granules may lower the overall treatment capacity of the reactor^13^,^14^. Thus, to ensure an efficient and stable operation of granule reactor, a better understanding of the granule properties is needed. Several previous studies have provided qualitative evaluation on the granule characteristics and corresponding treatment performances^15^,^16^. However, quantitative information is still lacking.

This work aims to provide quantitative evaluation on the characteristics of both activated sludge granules and flocs. For this purpose, a new methodology was developed by integrating fuzzy analytic hierarchy process (FAHP), accelerating genetic algorithm (AGA) and entropy weight method to link the sludge characteristics and the pollutant removal performances. FAHP is a process that simulates human being’s appraisal of ambiguity when complex multi-attribute decision making problems are encountered, and allows an accurate description of the decision making process^17^,^18^. To resolve the complex nonlinear calculation problems in the utilization of FAHP, accelerating genetic algorithm (AGA), a global search algorithm, could be used^19^. As an improvement of the genetic algorithm, the AGA successfully reduces the computational efforts and accelerates the convergence^19^.

Because of the subjectivity of the weight determined by the FAHP, the entropy weight method, an objective way for weight determination derived from information science, should be integrated with the FAHP approach. Information entropy, a measurement of the disorder degree of a system, can measure the amount of useful information with the data provided. A higher difference of the values among the evaluation indexes results in a greater entropy^20^. However, it depends on the difference of the evaluation index values only.

Therefore, given the complex characteristics of activated sludge and the limitations of the above-mentioned individual analytical/assessment techniques, here we developed a novel quantitative evaluation methodology through integrating FAHP, AGA and entropy weight method. With the FAHP and AGA, the subjective weights of the evaluation indexes for activated sludge granules and flocs could be determined. Then, the entropy weight approach is used to identify the objective weight of the evaluation indexes. As thus, the quantitative evaluation of granules and flocs could be performed. The integrated method developed here could provide a useful tool to guide the design and operation of granule-based wastewater treatment processes and might be extended for quantitative evaluations of various other biological processes.

## Results and Discussion

### Selection of evaluation samples and indexes and calculation of membership degrees

Table 1 lists the twelve evaluation samples, including eight types of activated sludge granules cultured in SBRs and four types of activated sludge flocs cultured in SBRs. The evaluation indexes of COD, TN and TP removal efficiencies, MLSS, SVI, size, settling velocity and stability are summarized in Table 1. The evaluation indexes of COD, TN and TP removal efficiencies were selected to evaluate the performance of simultaneous nitrogen and phosphorus removal. The SVI, size and settling velocity of sludge were chosen to compare their characteristics. To evaluate the reactor performance, the MLSS in reactors and the reactor stability were also considered as evaluation indexes.

**Figure 1:**
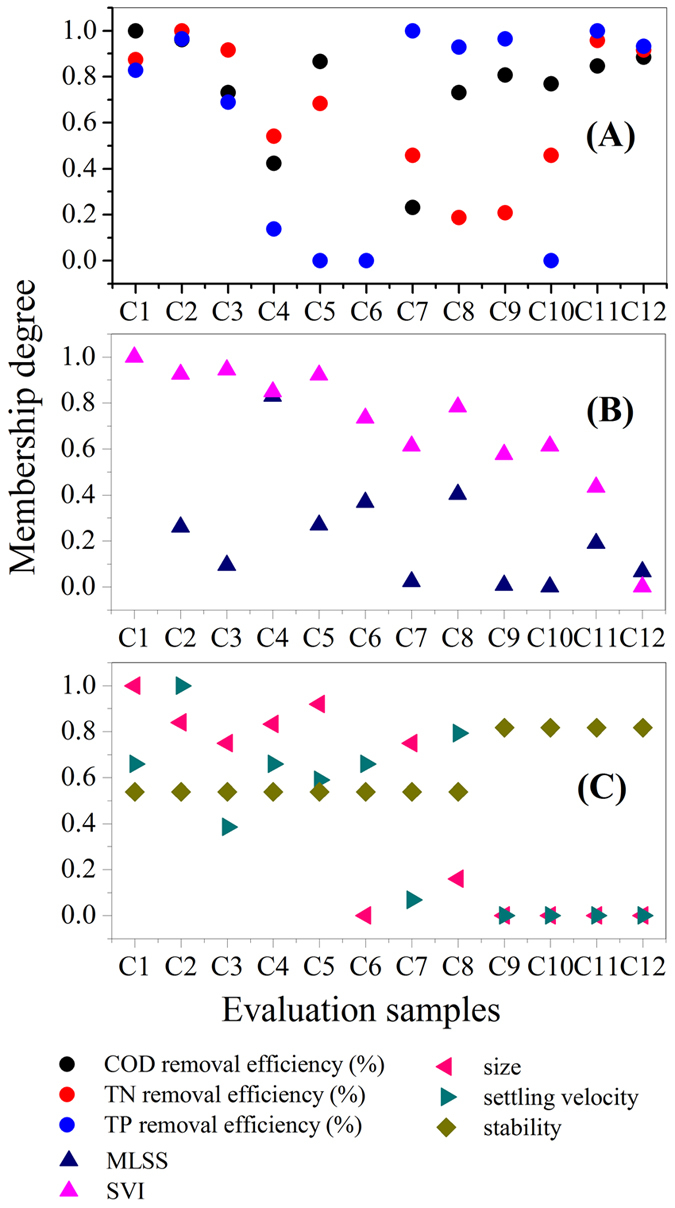
Membership degrees of the evaluation index.

For the first seven evaluation indexes, the membership degrees were calculated respectively using Eqs (2, 3, 4). The high values of COD, TN and TP removal efficiencies and MLSS indicate a good performance of the SBR systems, and high values of the settling velocity suggests good settling properties. Thus, the membership degrees of the five evaluation indexes were calculated using Eq. (2). On the contrary, the good settling capabilities of the flocs or granules were reflected by a lower SVI value. Thus, the membership degree of the SVI index was estimated by Eq. (3). Sludge size is one of the most important characteristics for flocs or granules. The size had a great influence on nitrogen removal by granules. A smaller granule diameter coincided with lower nitrogen removal efficiency, while at a larger granule diameter the granules started to break, resulting in big pores and flattened or kidney-shaped structures^4^. The structure and stability of granules were greatly related to the diffusivity of substrate and oxygen in granules^21^. Due to a diffusion limitation, the optimal diameter of granules in an SBR was suggested to be 1–3 mm^22^. In this study, the optimal value of the size was chosen as 1.3 mm^14^, and the membership degree of the evaluation index of granular size was calculated using Eq. 4. For the last evaluation index of stability, the fuzzy linguistic approach was used to compute the membership degree. The membership degree of the evaluation indexes are shown in Fig. 1.

### Weight determination of the evaluation indexes

The integrated weights of the evaluation indexes, denoting the importance of the evaluation indexes, were calculated by integrating the FAHP and the entropy weight approach.

First, the subjective weight of the evaluation index was obtained by the FAHP approach. Generally, the COD, TN and TP removal efficiencies were more important than the other five evaluation indexes. Also, the evaluation indexes of SVI and settling velocity were more important compared to those of MLSS, size and stability. Thus, the complementary judging matrix (*A*) was constructed as follows:


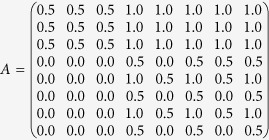


Secondly, by optimizing the objective function with the AGA, the subjective weights of the eight evaluation indexes and the consistency index coefficient (*CIC*(*m*)) were calculated. The calculated *CIC*(*m*) value of 0.117 was lower than that of the given critical *CIC*(8) of 0.232 (Table 2), indicating that the calculated weights of the evaluation indexes were reasonable. The subjective weights of the evaluation indexed gained by FAHP are listed in Table 3.

Thirdly, the objective weights of the evaluation indexes were obtained by the entropy weight approach and calculated using Eqs 6 and 7. The corresponding values are also listed in Table 3. To evaluate the activated sludge flocs and granules comprehensively, the integrated weights of the evaluation indexes were computed using Eq. 8 and the values are summarized in Table 3.

Generally, a high weight value means the greater importance of the evaluation index for the decision-making process. The results of the integrated weights of the eight evaluation indexes listed in Table 3 show that the weight of settling velocity was high. The high settling velocity could maintain the sludge in the reactor and it was a selection pressure for successful aerobic granulation^23^. The weight of COD removal efficiency was much lower than those of TN and TP removal efficiencies. The weight of SVI was also smaller than those of MLSS and particle size. Compared with other evaluation indexes, the stability was relatively less important because of the fluctuation of both granule- and floc-based SBR systems.

### Evaluation results

**Figure 2:**
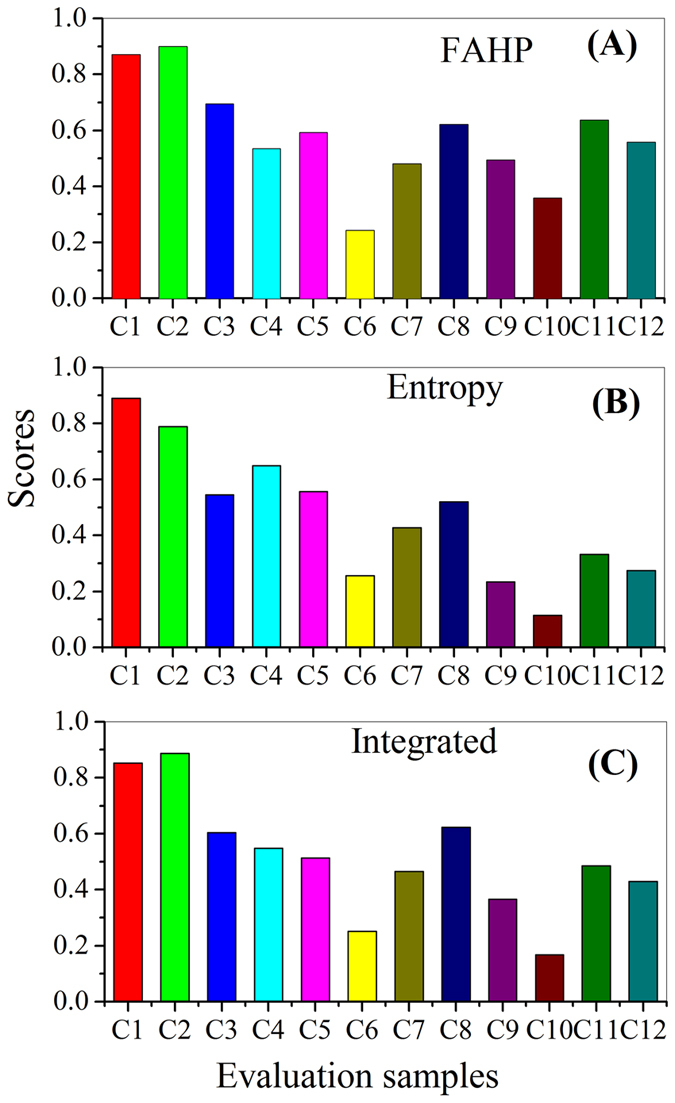
Evaluation results by the FAHP, entropy weight approach and the integrated method.

The scores of the evaluation samples by FAHP and entropy weight approach were respectively calculated (Fig. 2) and are given as follows:

*S*_*FAHP*_ = (0.871, 0.900, 0.695, 0.534, 0.592, 0.242, 0.481, 0.622, 0.494, 0.359, 0.638, 0.558).

*S*_*entropy*_ = (0.890, 0.788, 0.544, 0.649, 0.556, 0.255, 0.428, 0.520, 0.234, 0.114, 0.332, 0.275).

The evaluation results show that the calculated scores of granules were higher than those of flocs, suggesting that granules had more favorable characteristics than flocs. The integrated scores of the evaluation samples, with integration of FAHP and entropy approaches, were obtained using Eq. 9:

*S*_*integrated*_ = (0.853, 0.887, 0.604, 0.548, 0.513, 0.252, 0.465, 0.623, 0.366, 0.167, 0.485, 0.430).

The first eight values of *S*_*i*_ (except sample C6) were much higher than the other four values, indicating that the comprehensive characteristics of granules were better than those of flocs. The low integrated score of sample C6 was because the COD, TN and TP removal efficiencies of sample C6 were lower than those of other samples. On the other hand, the particle size of C6 was higher than those of other granules. A large particle size could increase the mass transfer limitation. Thus, the membership degrees of COD, TN and TP removal efficiencies and particle size of sample C6 were lower. However, the weights of TN and TP removal efficiencies and particle size of sample C6 were relatively higher. Thus, the integrated score of sample C6 was lower.

As shown in Table 1, the capabilities of flocs and granules for simultaneous nitrogen and phosphorus removal differed slightly, although the size of granules was much larger than that of flocs. The larger size of granules increased the biomass retention and favored a higher settling velocity and a lower SVI compared to the flocs, but it also increased the mass transfer limitation and may impair the long-term operating stability because the microorganisms in the granule center would undergo microbial decay or lysis under substrate deficiency^21^. Thus, an optimized control of granule size is essential for maintaining good pollutant removal performance and long-term stability of granule-based reactors^21^.

Our evaluation results are in consistent with those reported previously. Pronk *et al*.^6^ investigated the operation of one of the currently largest full scale aerobic granular sludge plants treating domestic sewage and found that both energy usage and specific volume of aerobic granular sludge plants were lower than those of the conventional activated sludge plants with comparable or better effluent quality. Additionally, for textile wastewater treatment, higher anaerobic and overall COD removal efficiencies and better detoxification potentials were observed for granule-based reactors compared with floc-based reactors^24^. Furthermore, when the performance of a granular sludge system was compared with the a full-scale wastewater treatment plant to treat mixed a municipal-textile wastewater, the granular sludge system was found to be able to produce an effluent of comparable quality with a simpler treatment scheme, a much lower hydraulic retention time and a lower sludge production^25^. These results demonstrate that aerobic granular sludge can be more effectively implemented for the treatment of various wastewaters.

The integrated method developed in this work has not been used to evaluate the biological wastewater treatment systems. Such an approach method gives a solution for the comprehensive evaluation of the characteristics of activated sludge granules and flocs, and can also be used for evaluating and comparing other similar systems. This approach can provide useful information for the application of activated sludge granules in full-scale wastewater treatment plants.

## Methods

In this study, four types of activated sludge flocs and eight types of aerobic granules were evaluated. The data sets from the reported experimental results are summarized in Table 1. In the first eight systems, aerobic granules were used to treat a nutrient-rich synthetic wastewater and industrial wastewater for simultaneous nitrification, denitrification and phosphorus removal^4^,^7^,^10^,^11^,^26^,^27^,^28^,^29^. The other four systems were floc-based SBRs for synthetic wastewater and slaughterhouse wastewater treatment^30^,^31^,^32^,^33^. Because of the incomplete experimental data reported in literature above, the experimental results of Su and Yu^16^ were also used for evaluation.

### Model establishment

A new methodology with an integration of FAHP, AGA and entropy weight method was established to quantitatively evaluate and compare the characteristics of different sludge samples.

First, the evaluation samples and evaluation index were selected. In this work, *n* of evaluation samples and *m* types of evaluation indexes were chosen. As listed in Table 1, the chemical oxygen demand (COD) removal efficiency, total nitrogen (TN) removal efficiency, total phosphorus (TP) removal efficiency, mixed liquor suspended solids (MLSS), sludge volume index (SVI), size, settling velocity and stability were selected as the evaluation indexes and represented using the following equation.


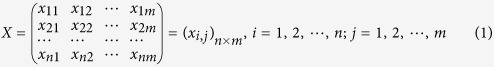


where *x*_*ij*_ represents the *j*th evaluation index of the *i*th sample.

For the first seven evaluation indexes, they can be expressed with the real numbers. But for the last evaluation index (stability), it was difficult to express in a quantitative form. To solve this problem, the fuzzy linguistic approach was used to express the evaluation index of stability. The fuzzy linguistic approach is an approximate technique to deal with the fuzzy and unrigorous qualitative aspects of problems^34^. For this approach, 5–11 linguistic scales are usually used to incorporate the expert judgments^35^. In this work, five linguistic scales, i.e., bad (B), poor (P), fair (F), good (G), excellent (E), were considered for the qualitative expression of the evaluation indexes of stability^36^.

After the selection of the evaluation samples and indexes, the membership degree of the evaluation samples was calculated. Because of the different characteristics of the evaluation indexes, the membership degrees of the evaluation indexes were computed using different approaches.

If the evaluation index is the-larger-the-better, it can then be calculated using the following equation:


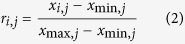


If the evaluation index is the-small-the-better, it can then be expressed as:


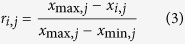


If the evaluation index is nominal-the-better, it can then be expressed as:


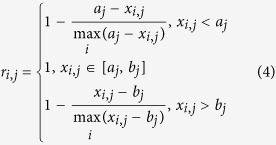


where *max* and *min* are the maximum and minimum function respectively; [*a*_*j*_, *b*_*j*_] is the best fitting interval of the *j*th index; *r*_*i,j*_ is the relative membership degree of the *j*th evaluation index of the *i*th sample.

**Figure 3:**
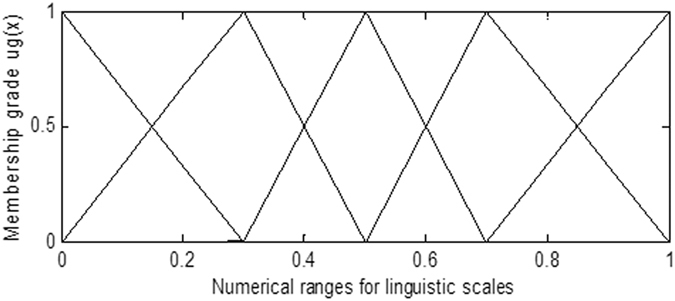
Membership spread of the linguistic variables.

For the evaluation indexes of COD, TN and TP removal efficiency, MLSS, SVI, size and settling velocity, their membership degrees could be computed using Eqs 2, 3, 4. However, these equations were not suitable to calculate the membership degree of the last evaluation index, i.e., stability, because of its expression with the fuzzy linguistic approach. Therefore, the membership degree of stability was identified according to Chowdhury and Husain^36^, as shown in Fig. 3.

Determination of the weights of evaluation indexes, including both subjective and objective weights, is of critical importance. The subjective weight of the evaluation index could be determined by the knowledge or experience of experts. However, the judgment of an expert can only reflect the facts of the complicated objects to some degree^37^. The objective weights could be determined only depending on the difference of the data sets. Hence, to improve the reliability of the evaluation results, the integration of a subjective weight determination approach, i.e., FAHP, and an objective weight determination approach, i.e., entropy weight approach, was used to identify the weight of the evaluation indexes.

The subjective weight with FAHP was calculated with the method in our previous study^19^. In brief, a fuzzy complementary judging matrix *A (a*_ij_), which was used to calculate the value of *CIC*(*m*), was first established. After the construction of the matrix *A*, the subjective weights of the evaluation indexes were calculated by optimizing the following objective function with the AGA.


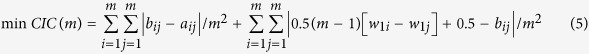



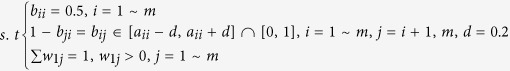


where *b*_ij_ is the optimum fuzzy consistency modified judging matrix of matrix *a*_ij_, and *w*_1j_ are the objective weights of the evaluation indexes.

Then, the consistency was examined with the consistency index coefficient (*CIC*(*m*)), in which *m* is the number of the evaluation index. The subjective weights of the evaluation indexes could be determined until the calculated *CIC*(*m*) value is less than given critical values.

The objective weight of the evaluation index was calculated using the entropy weight approach. Information entropy, derived from thermodynamics and used to describe the irreversible phenomenon of a motion or a process, is a criterion for the amount of uncertainty represented by a discrete probability distribution^38^. A narrowed distribution represents less uncertainty than a broad distribution. Therefore, the entropy could be used to calculate the weight of each evaluation index. When the difference of the values among the evaluation samples is higher, the entropy becomes smaller, indicating that this evaluation index provides more useful information. Thus, the weight of the evaluation samples is higher^20^. The entropy values can be calculated with the following equation:


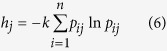


where *k* = 1/ln (*n*), 
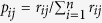
.

Then, the objective weight value *w*_*2j*_ of the evaluation index is:


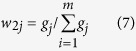


where *g*_*j*_ = 1 − *h*_*j*_.

After both subjective and objective weights were calculated with the FAHP and the entropy weight approaches, respectively, the integrated weight of the evaluation index coupling subjective and objective weights could be computed using the following equation:


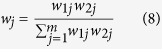


where *w*_*1j*_ is the subjective weight of the *j*th evaluation index, and *w*_*2j*_ is the objective weight of the *j*th evaluation index, *w*_*j*_ is the integration weight of the *j*th evaluation index.

Finally, the evaluation results of the activated sludge flocs and granules were obtained using Eq. 8:


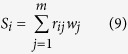


where *r*_*ij*_ is the membership degree of the *j*th evaluation index of the *i*th evaluation samples, *S*_*i*_ is the score of the evaluation sample.

**Figure 4:**
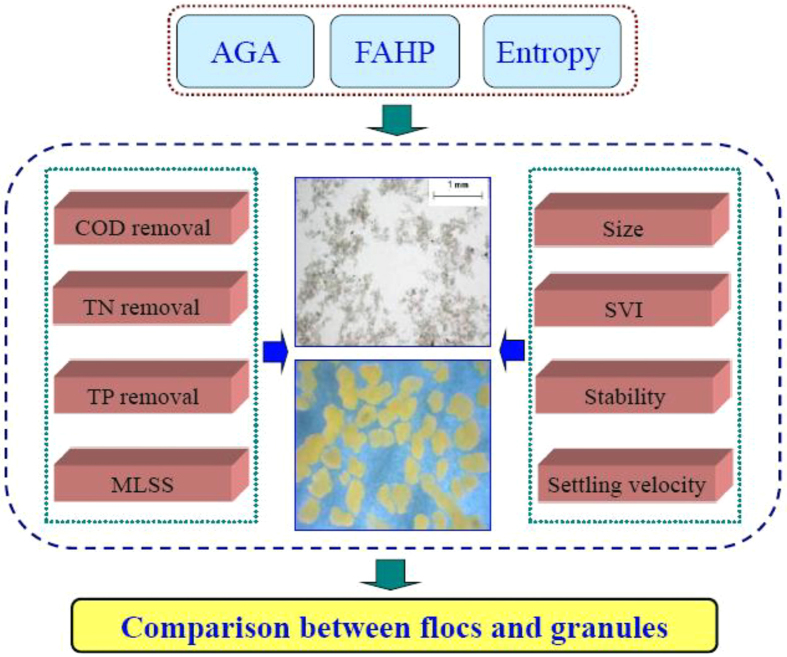
Flowchart of the evaluation model.

With the obtained *S*_*i*_, the activated sludge flocs and granules could be compared and evaluated. A higher value of *S*_*i*_ indicates the better performance of the evaluation sample. The evaluation procedure for the activated sludge flocs and granules is illustrated in Fig. 4.

## Conclusions

A novel methodology with integration of FAHP, AGA and entropy weight approaches was established to quantitatively evaluate the characteristics of activated sludge granules and flocs in SBRs for simultaneous carbon, nitrogen and phosphorus removal. The evaluation gave different main scores for the tested flocs and granules. The higher scores of granules suggest that granules possess more favorable overall characteristics than flocs. Thus, this integrated methodology may provide a useful tool for guiding the design and operation of granule-based wastewater treatment processes as well as for quantitative evaluations of various biological processes.

## Additional Information

**How to cite this article:** Fang, F. *et al*. Quantitative evaluation on the characteristics of activated sludge granules and flocs using a fuzzy entropy-based approach. *Sci. Rep.* **7**, 42910; doi: 10.1038/srep42910 (2017).

**Publisher's note:** Springer Nature remains neutral with regard to jurisdictional claims in published maps and institutional affiliations.

## Figures and Tables

**Table 1:** Experimental data sets.

Index (*Xi*)	COD removal efficiency (%) (*X*_*1*_)	TN removal efficiency (%) (*X*_*2*_)	TP removal efficiency (%)(*X*_*3*_)	MLSS (mg/L) (*X*_*4*_)	SVI (L/mg) (*X*_*5*_)	Size (mm) (*X*_*6*_)	Settling velocity (m/h) (*X*_*7*_)	Stability (*X*_*8*_)	Reference
C1	100	94	94	23600	14	1.3	36*	Fair	4
C2	99	97	98	8000	22	1.7	51	Fair	10
C3	93	95	90	4500	20	1.0	24	Fair	7
C4	85	86	74	20000	30*	1.1	36*	Fair	11
C5	96.5	89.4	/	8200	22.3	1.5	33	Fair	29
C6	74	73	70	10270	42.1	3.5–4.1	36*	Fair	28
C7	80	84	99	3000	50–60	1	10	Fair	27
C8	90.6–95.4	72.8–82.1	95.8–97.9	11000	37	3.4	18.6–65.1	Fair	26
C9	95	78	98	2670	59	0.1*	7*	Excellent	31
C10	94	84	70	2500	55	0.1*	7*	Excellent	30
C11	96	96	99	6500	74*	0.1*	7*	Excellent	32
C12	97	95	97	3900	120	0.1*	7*	Excellent	33

**Table 2:** Consistency index of FAHP.

*m*	Consistency index coefficient *CIC*(*m*)
3	0.185
4	0.196
5	0.236
6	0.243
7	0.224
8	0.232
9	0.234

**Table 3:** Weights of the evaluation index.

Index	*w*_*1j*_	*w*_*2j*_	*w*_*j*_
COD removal efficiency (%) (*X*_*1*_)	0.186	0.055	0.091
TN removal efficiency (%) (*X*_*2*_)	0.186	0.078	0.129
TP removal efficiency (%)(*X*_*3*_)	0.186	0.143	0.237
MLSS (mg/L) (*X*_*4*_)	0.054	0.213	0.103
SVI (L/mg) (*X*_*5*_)	0.138	0.046	0.057
Size (mm) (*X*_*6*_)	0.055	0.246	0.121
Settling velocity (m/h) (*X*_*7*_)	0.138	0.210	0.258
Stability (*X*_*8*_)	0.056	0.009	0.004
	**FAHP**	**Entropy**	**Integrated**
